# Effect of red beetroot juice on oxidative status and islet insulin release in adult male rats

**DOI:** 10.1186/s13098-022-00830-z

**Published:** 2022-04-23

**Authors:** Armin Sayyar, Mohammad Oladi, Mehran Hosseini, Samaneh Nakhaee, Zomorrod Ataie, Khadijeh Farrokhfall

**Affiliations:** 1 Student Research Committee, Birjand University of Medical Sciences, Birjand, Iran; 2 Cellular and Molecular Research Center, Birjand University of Medical Sciences, Birjand, Iran; 3 Medical Toxicology and Drug Abuse Research Center (MTDRC), Birjand University of Medical Sciences, Birjand, Iran; 4 Health Clinical Science Research Center, Islamic Azad University, Zahedan Branch, Zahedan, Iran; 5 Student Research Committee, Islamic Azad University, Zahedan Branch, Zahedan, Iran; 6 Experimental Medicine Laboratory, Cardiovascular Diseases Research Center, Birjand University of Medical Sciences, Birjand, Iran

**Keywords:** Beetroot, Islet insulin secretion, Malondialdehyde, Nitric oxide

## Abstract

**Introduction:**

Beetroot is rich in inorganic nitrate and it has been shown that inorganic nitrate has beneficial effects on metabolic syndrome. This study aims to investigate the effect of red beetroot juice (RBJ) on carbohydrate metabolism in adult insulin-resistant rats.

**Materials and methods:**

Sixteen male Wistar rats (32 weeks old) were divided into two equal groups: control and RBJ. Treatment with drinking water (control) and 100% RBJ (RBJ) was lasted for 5 weeks. At the end of the 4th week the intraperitoneal glucose tolerance test was performed and at the end of the study period animals were sacrificed and blood and tissue (aorta, heart, and liver) samples were collected. Furthermore, pancreatic islets were isolated and their insulin secretion activity was investigated in different glycemic conditions.

**Results:**

Compared to the control group, RBJ-treated rats showed lower blood glucose and insulin levels in the glucose tolerance test. Serum and tissue levels of nitric oxide in the RBJ group were significantly higher than those in the control group. The liver peroxidation and serum aspartate transaminase levels were significantly increased in the RBJ-treated animals compared to the control group. The islets of RBJ group exhibited lower insulin secretion, especially in 16.7 mM glucose concentration (supraphysiologic condition) than control group.

**Conclusions:**

RBJ consumption improves glucose metabolism in rats via increasing nitric oxide metabolites in an insulin-independent manner. However, future studies are needed to minimize the potential hepatic adverse consequences.

**Supplementary information:**

The online version contains supplementary material available at 10.1186/s13098-022-00830-z.

## Introduction

Insulin resistance (IR) is a pathologic condition when cells of the body do not adequately respond to the insulin hormone. In consequence, the blood glucose and insulin levels are elevated in the IR patients. The IR is closely associated with metabolic syndrome because it negatively affects blood pressure, carbohydrate and lipid metabolisms, and body weight [1]. IR incidence tends to increase with aging, and as the number of older people globally increases; thereupon, the global prevalence of IR is rising [2].

Lifestyle and environmental factors are the prominent participants in the IR condition. Several dietary interventions have been shown to decrease IR. Currently, diet intervention is recommended for people with IR [3, 4]. Red beetroot (*Beta vulgaris rubra*) is an important source of inorganic nitrate (NO_3_), and its consumption increases the bioavailability of nitric oxide (NO) in the body. Since in some pathological conditions such as IR and hypertension, the bioavailability of NO is reduced, it seems that the consumption of a nitrate-rich diet can probably be effective in controlling these conditions [5–7]. Beetroot consists of various minerals, including potassium, sodium, phosphorous, calcium, magnesium, copper, iron, zinc, and manganese, and biologically active compounds such as betalains, saponins, polyphenols, flavonoids [8]. Red beetroot is a rich source of red pigments known as betalains [9]. The two main constituents of betalains are the red-violet betacyanins and the yellow betaxanthins. The most abundant and primary betacyanin of red beetroot is betanin, which is responsible for 75–95% of the total beetroot color [10]. Betalains have diverse bioactivities, including antioxidant, anti-inflammatory, immune-modulatory, and cancer chemoprotective properties [11]. Red beetroot has been ranked among the ten most potent antioxidant vegetables. The antioxidant capacity of red beetroot is closely associated with the betalains content [9].

Previous studies demonstrated several health benefits of beetroot on age related diseases include hypertension, diabetes and hypercholesterolemia [6, 7, 12]. The beneficial effects of beetroot have been attributed to the presence of high amounts of NO_3_ and its effects on nitrate–nitrite–nitric oxide (NO) pathway.

## Materials and methods

### Animals and diets

Sixteen male Wistar rats (32 weeks old) were obtained from the Research Centre of Experimental Medicine, Birjand University of Medical Sciences, Iran. All experiments were conducted in accordance with standard ethical guidelines, and the local ethics committee approved the study (No: IR.BUMS.REC.1396.206). The animals were housed (n = 2 per cage) under the standard condition (12 h light/dark cycle, 24 °C and 25–30% relative humidity) throughout the experimental period.

The red beetroot was purchased from the local market of Mashhad, Iran and then its species and genus were approved by the Herbarium of the Plant Sciences Research Institute of Ferdowsi University of Mashhad (No: E1254-FUMH). The RBJ was freshly obtained from *Beta vulgaris* rubra without any chemical additives. The nitrate and carbohydrate contents of the RBJ were determined by standard protocols in institute of standards and industrial research of Iran. Accordingly, the RBJ contained 570.5 mg/1000 g (1096 mL) nitrate and 2.52 g% (w/v) carbohydrates.

### Experimental procedures

After 2 weeks of acclimatization, adult rats were weighed and randomly assigned into two groups (*n* = 8 per group). The control group received tap water as drinking water, while the investigation group received 100% RBJ during the study period (5-week).

### Intraperitoneal glucose tolerance test (IPGTT)

At the end of the 4th week of the study and after an overnight fasting (12–14 h), the IPGTT was performed under pentobarbital anesthesia (60 mg/kg, IP). After obtaining the first blood sample from the tail vein at time zero, each rat received a single IP dose of 2 g/kg of glucose solution (50% w/v, 0.4 mL/100 g body weight). Blood samples (0.3 mL each) were taken from the tail vein at 15, 30, 60, 90 and 120 min into heparinized tubes for glucose and insulin measurements [13]. Blood samples were centrifuged immediately (3000×*g*; 10 min, 4 °C), and then plasma was separated and stored at − 20 °C until analysis. Homeostasis model assessment of insulin resistance (HOMA-IR) $\left \lceil {\text{HOMA-IR}}=\frac{\left(\text{Glucose}(\text{mmol}/\text {L})\times {\text{Insulin}}\; \text{}({\upmu}{\text{U}}/\text{mL})\right)}{22.5} \right\rceil$ was calculated.

### Measurement of systolic blood pressure (SBP)

At the end of the study (after 5 weeks), the non-invasive systolic blood pressure was measured using a Piezo-Electric Pulse Transducer (AD Instruments, Australia) and an inflatable tail-cuff connected to a transducer recording pressure and PowerLab data acquisition unit (AD Instruments) in conscious animals. Animals were acclimatized in the restrainer chamber for 15 min per day for a period of 3 days before the starting of the SBP evaluation. After that, the trained animals were placed in a restrainer chamber and allowed to acclimatize with the chamber for 15 min. During the acclimation period their tails were warmed by raising the water bath (37 °C). Then, an occlusion and sensor cuff was placed around the proximal portion of the tail and several recordings of BP were performed. The mean of the five stable consecutive records (~ 1 min interval) was calculated for each animal [14].

### Blood and tissues sampling

Following SBP measurement and 12–14 h fasting, animals were anesthetized using pentobarbital (60 mg/kg, IP) and blood was taken from the heart. Three mL of the blood samples were transferred in ethylene diamine tetra-acetic acid (EDTA) tubes for hematological evaluation and the remained volume (5–6 mL) was transferred into tubes containing no anticoagulants to collect blood serum. Blood samples were centrifuged at 3000×*g* for 10 min, and then serum was separated and stored at − 20 °C until biochemical assessments.

Following blood collection, the heart, aorta, and liver were dissected out and stored at − 80 °C. Tissue samples were homogenized in ice-cold PBS (phosphate buffered saline, Ph ≈ 7.4, 1:10) by a homogenizer (Miccra D-1, Germany). Then, tissue homogenates were centrifuged (15,000×*g* for 20 min at 4 °C) and supernatants were collected at − 20 °C.

### Biochemical assessments

#### Assessment of NO metabolites

Serum and tissue levels of NO metabolites (NOx) were evaluated by the Griess reaction method as previously described [15]. In brief, serum and supernatant samples were deproteinized by zinc sulfate (15 mg/mL). Then, 100 µL of deproteinized sample and 100 µL of vanadium chloride (saturated solution 0.8% in HCl 1 molar) were transferred in to each well of a 96-well microplate. Eventually, 100 µL of the Griess reagent (sulfanilamide solution 0.2% in HCl 5% and NEDD solution 0.1% in distilled water) was added to each well and the plate was incubated for 30 min at 37 °C. Finally, the absorbance was read in 540 nm. NOx concentration was determined from the linear standard curve established by 0–80 µmol sodium nitrate.

#### Assessment of lipid peroxidation

Lipid peroxidation in the tissue homogenates and serum samples were determined by measuring the amounts of malondialdehyde (MDA), the end product of the lipid peroxidation process [16]. In brief, 100 µL of each sample (serum or homogenate supernatant) was added to 200 µL thiobarbituric acid (0.67%) and 600 µL of phosphoric acid (1%). Then, the mixture was incubated at 90–100 °C for 45 min and the reaction was stopped by placing sample tubes on ice. After cooling, the *n*-butanol (800 µL) was added to each tube and vigorously mixed. Then, it was centrifuged at 5000 rpm for 10 min. The resulting supernatant was removed (200 µL) and its optical absorbance was measured at 532 nm. The concentration of MDA was determined from the linear standard curve obtained by 0–40 µmol of 1,1,3,3-tetraethoxypropane.

#### Other biochemical and hematological assessments

Insulin level was measured by insulin ELISA kit (insulin; mercodia, Sweden). Plasma glucose was evaluated by the glucose oxides method (Pars azemun Co., Iran). Lipid profile [triacylglycerol (TAG), total cholesterol (TC), low-density lipoprotein (LDL-C), high-density lipoprotein (HDL-C)] and liver enzymes [aspartate transaminase (AST) and alanine transaminase (ALT)] were measured using an autoanalyzer machine (Integra, Germany) and Roche diagnostic kits (Mannheim, Germany). Hematological parameters were measured by a human’s automated hematology analyzer (Sysmex, Germany). The intra-assay coefficient of variation for insulin, glucose, NOx, and MDA was 3.41% ± 0.5, 2.15 ± 0.3, 2.01 ± 0.12, and 4.69 ± 1.02, respectively.

### Islet isolation and glucose stimulated insulin secretion (GSIS)

Following blood collection pancreatic islets were isolated by intrapancreatic duct injection of Hanks’ balanced salt solution (HBSS) [pH, 7.4; containing NaCl, 136; KCl, 5.36; CaCl_2_, 1.26; MgSO_4_·7H_2_O, 0.8; Na_2_HPO_4_·2H_2_O, 0.33; KH_2_PO_4_ 0.44; NaHCO_3_, 4.16 all in mM (Merck, Germany)] containing 0.5 mg/mL of collagenase P (Roche, Cat. #1213, Germany). After digestion and washing, the islets were hand-picked under a stereomicroscope [13]. For evaluation of in vitro insulin secretion, batches of 5 islets (three or four replications for each condition from 4 animals) were transferred into a 6-well plastic plate, containing 1 mL of Krebs–Ringer solution [(pH, 7.4); NaCl 115; KCl 5; MgCl_2_ 6H_2_O 1; CaCl_2_ 2.5; NaHCO_3_ 24 (Merck, Germany); HEPES, 16 (Sigma, USA) all in mM] and 0.5 g/dL BSA (Fluka, USA) [17]. Then, different glucose concentrations (5.6, 8.3, 16.7 mM) were added to each well. To investigate the role of NO, the incubation mediums of six wells containing 16.7 mM glucose in each group were supplied by nitric oxide synthase (NOS) inhibitor; aminoguanidine (AG, 10 mM). All plates were incubated for 60 min in a CO_2_ incubator at 37 °C and gassed with 95% O_2_/5% CO_2_. After slightly shacking, the aliquots of supernatant were collected under a stereomicroscope and stored at − 20 °C for insulin determination [18].

### Statistical analysis

All analyses were validated by D’Agostino and Pearson (omnibus K2 test performed with Prism version 5) normality test. All data are expressed as mean ± SEM, and data on IPGTT were analyzed by 2-way ANOVA followed by a Bonferroni post-hoc test and the results of the other tests were compared by t-test using Graph Pad Prism software (Version 5). The glucose and insulin concentrations were assessed by calculating the area under the curve (AUC) of IPGTT. Because of the skewed distribution of NOx and MDA values in the aorta, heart, and liver, non-parametric statistics were used, and data were presented as median (interquartile range). Mann–Whitney U test was used for comparison. All graphs were achieved using Graph Pad Prism software (Version 5). A value of *p <* 0.05 was considered statistically significant.

## Results

No death occurred during the study period. The mean daily water and RBJ intakes were 41.55 ± 6.75 and 56.96 ± 13.42 mL, respectively (Table 1). Accordingly, the animals of the RBJ group received approximately 30 mg of nitrate per day.

**Table 1:** Weight gain, hemodynamic and metabolic parameters in plasma of experimental groups

Variables	Groups	
	Control group	RBJ group
Weight gain (g/4 weeks)	13.50 ± 3.22	3.17 ± 4.86*
Daily swallow (mL/day/rat)^a^	41.55 ± 6.75	56.96 ± 13.42
SBP (mmHg)	104.46 ± 2.76	90.34 ± 3.69
Pulse rate (beat/min)	276.1 ± 29.77	301.3 ± 8.11
Fasting plasma glucose (mg/dL)	117.0 ± 2.72	100.6 ± 3.54**
Fasting plasma insulin (Pmol/L)	248.8 ± 21.58	49.20 ± 5.91***
AST (SGOT)	61.00 ± 1.29	102.3 ± 12.03*
ALT (SGPT)	58.25 ± 4.50	58.50 ± 2.09

Statistical comparison between groups was made using a t-test, values are mean ± SEM, n = 6 each group

*SBP* systolic blood pressure, *MDA* malondialdehyde, *NOx* nitrite + nitrate, *SGOT* serum glutamic-oxaloacetic transaminase, *AST* aspartate aminotransferase, *ALT* alanine aminotransferase, *SGPT* serum glutamic-pyruvic transaminase, *RBJ* red beetroot juice

*P < 0.05, **P < 0.01, ***P < 0.001, statistically significant differences between groups in the outlined sites. Sampling was conducted on fasting

^a^Values were approximately calculated

### Effect of RBJ on weight gain and blood pressure

The results are summarized in Table 1. At the beginning of the study, the mean ± SD of body weight of the control and the RBJ groups were 356.43 ± 11.35 g and 346.85 ± 12.59 g, respectively (P = 0.891). However, at the end of the study, the control group had higher body weight than the RBJ group (p = 0.045).

The results of SBP showed that there was no significant difference between the studied groups.

### Effect on liver enzymes

Compared with rats in the normal control group, RBJ consumption significantly (p < 0.001) elevated AST level but had no significant effect on ALT.

### Effects on glucose tolerance and insulin resistance

Fasting plasma glucose (FPG) levels in the control group were significantly higher than the RBJ (*P* < 0.01, Table 1).The IPGTT was performed to assess glucose tolerance in rats after 4 weeks intervention. No statistical difference in AUC of IPGGT curve was found between the studied groups (Table 2).

**Table 2:** Insulin resistance indices and variation plasma glucose and insulin following IPGTT

Variables	Groups		
	Control group	RBJ group	P value
HOMA-IR1	10.63 ± 1.01	2.056 ± 0.27***	< 0.0001
Glucose AUC (mmol/L/120 min)	956.8 ± 53.85	898.4 ± 17.77	0.327
Insulin AUC (Pmol/L/120 min)	64,870 ± 3953	30,100 ± 5794***	0.0009

Statistical comparison between groups was made using a t-test, n = 6 each group, values are means ± SEM

HOMA-IR: homeostasis model assessment of insulin resistance; RBJ: red beetroot juice, variations of plasma glucose and insulin concentrations are presented as the area under the curve (AUC) during IPGTT in different experimental groups. Sampling for HOMA-IR was given in fasting

***P < 0.001, statistically significant differences between Control and RBJ in the outlined sites

The HOMA-IR index (HOMA-IR > 2.5) of the control group was significantly higher than the RBJ group (*P* < 0.001, Table 2).

**Fig. 1:**
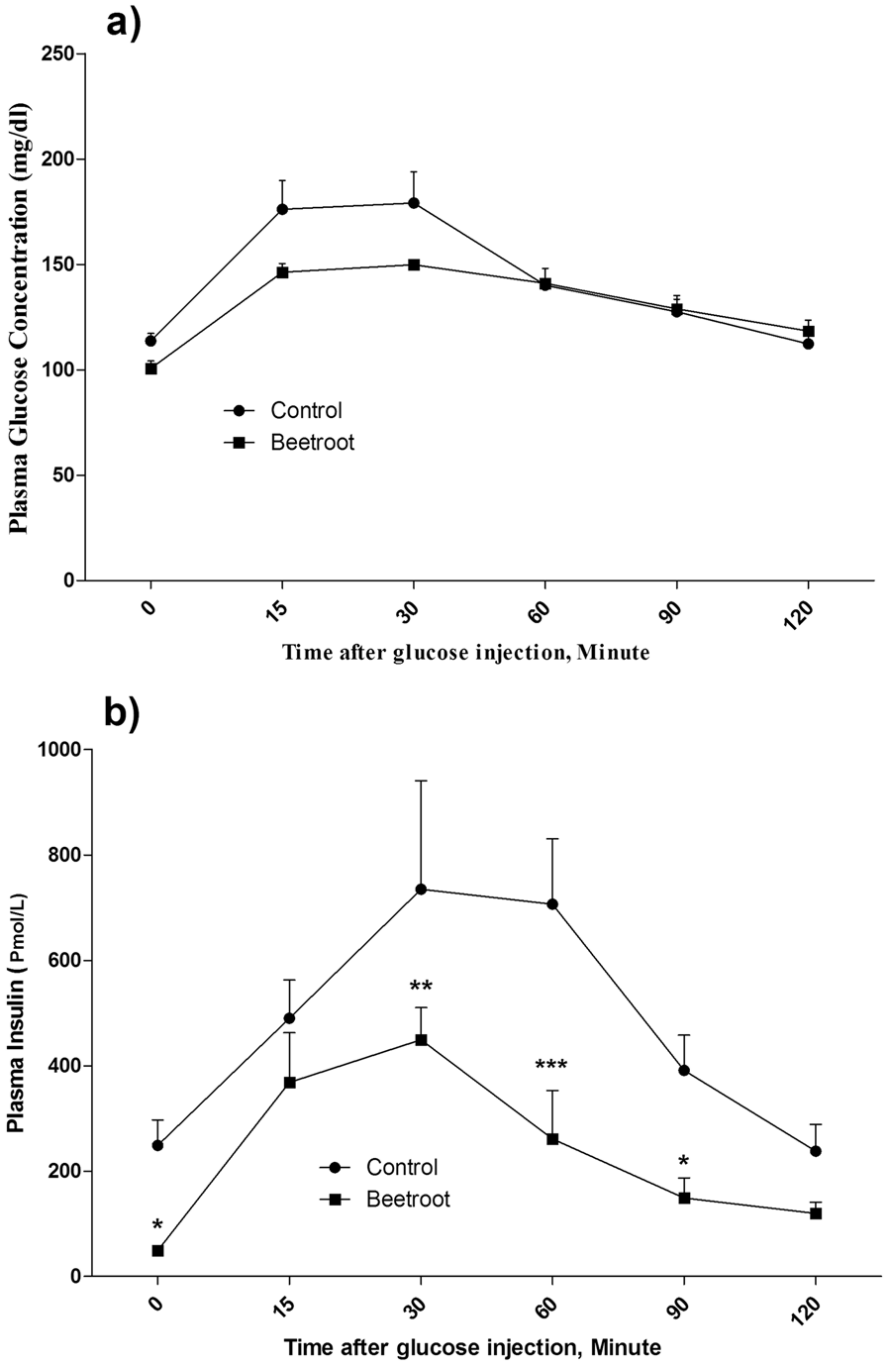
Intraperitoneal glucose tolerance test in animals:** a** plasma glucose concentration and **b** insulin level. Values are mean ± SEM. Statistical comparison between groups was made using two-way ANOVA and followed by a Bonferroni post-hoc test, n = 6 each group; Beetroot (red beetroot juice) vs. control (*P < 0.05, **P < 0.01, ***P < 0.001). The experiment was conducted on fasting

As shown in Fig. 1b, the basal insulin level in the control animals was significantly higher than in the RBJ group. Moreover, the means plasma insulin concentrations of RBJ group were lower than the control group at 30-, 60- and 90-min following glucose administration [min 30 insulin: 449.5 ± 60.44 vs. 734.6 ± 91.83 pmol/L, *P* < 0.01; min 60 insulin: 261.3 ± 91.56 vs. 706.3 ± 55.55 pmol/L, *P* < 0.001; min 90 insulin: 148.8 ± 38.30 vs. 391.0 ± 25.86 pmol/L, *P* < 0.05].

In consequence, the AUC of the plasma insulin concentration of the RBJ group was significantly lower than that of the control group (p < 0.001, Table 2).

### Effect on pancreatic insulin secretion

**Fig. 2:**
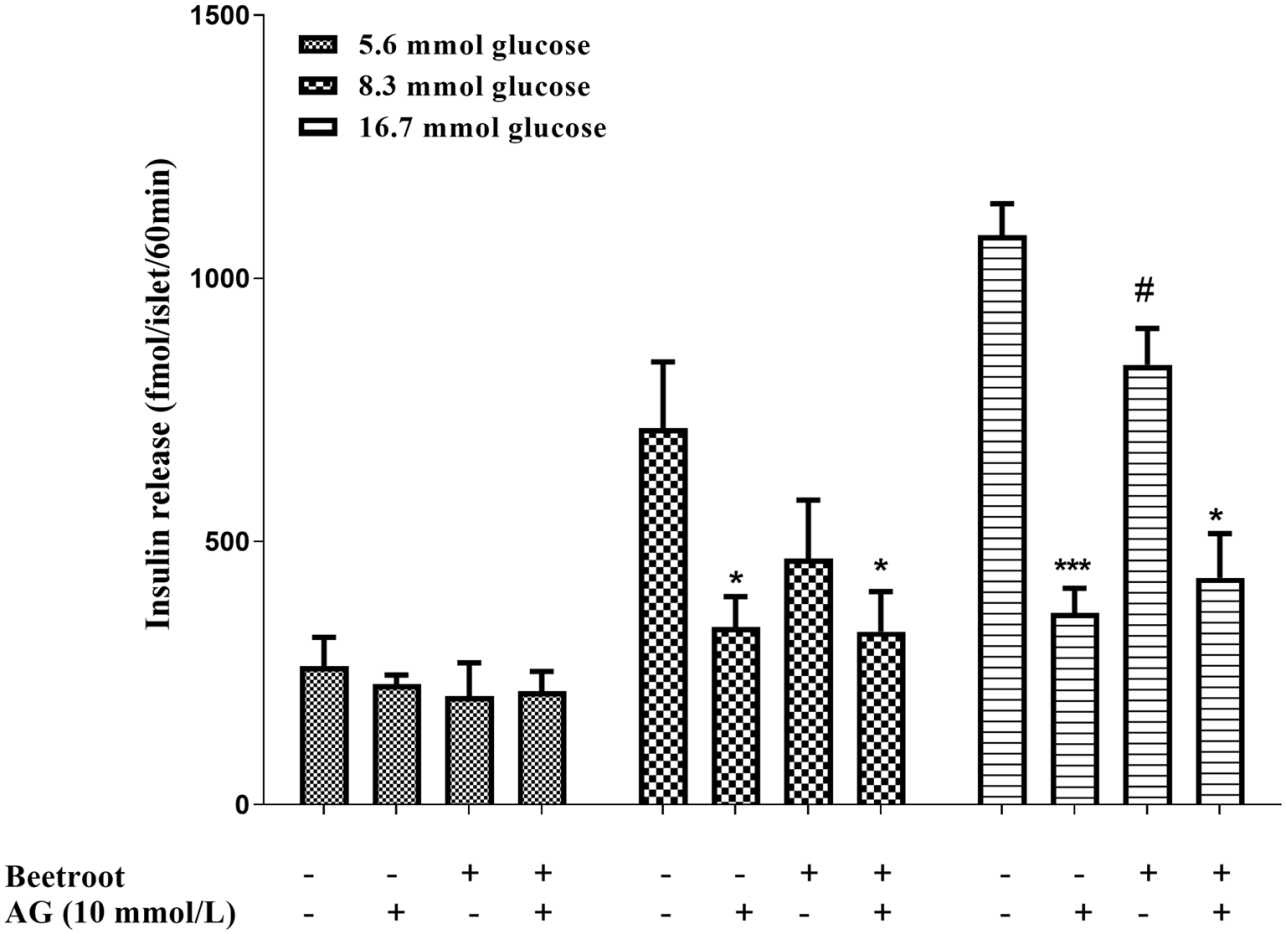
GSIS assay on isolated islets and the effect of nitric oxide inhibition on islet insulin secretion from experimental groups. Insulin release was measured during 1 h from groups of 5 islets at increasing glucose concentrations (5.6–16.8 mmol/L) after overnight fasting. Nitric oxide production was inhibited by Aminoguanidine (AG). Isolated islets were incubated in vitro with AG and 8.3 mM glucose followed by stimulation with 16.7 mM glucose. Insulin release was measured during 1 h from groups of 5 islets after overnight fasting. Values are mean ± SEM for 12 cups (3 cups each containing 5 islets for each condition from each animal; 4 animals in each group). Statistical comparison between groups in each glucose concentration was made using t-test. Beetroot (red beetroot juice) vs. without AG in same group (*P < 0.05, ***P < 0.001), Beetroot (red beetroot juice) vs. control (^#^*P* < 0.05)

The results indicated that the pancreatic insulin secretion in the RBJ group was lower (p < 0.05) than control group at the supraphysiological concentration (16.7 mM/L) glucose concentration (Fig. 2).

To investigate the role of NO in the suppression of pancreatic insulin secretion observed in the RBJ islets, we used a NOS inhibitor (AG). We found that, AG decreased insulin production in the control islets at both postprandial glucose concentration (8.3 mM/L) and supraphysiological concentration (16.7 mM/L) (p < 0.001). Incubation of RBJ islets with AG resulted in a decrease in insulin release only at supraphysiological concentration (16.7 mM/L) (p < 0.05, Fig. 2).

Inhibition of endogen nitric oxide decreased insulin release in control group more than RBJ group [49.91% ± 6.4% vs. 31.16% ± 2.8% at 8.3 mM/L glucose (p < 0.05) and 64.40% ± 2.9% vs. 50.51% ± 5.2% (p < 0.05) at 16.7 mM/L glucose].

### Effects on NOx and MDA

**Fig. 3:**
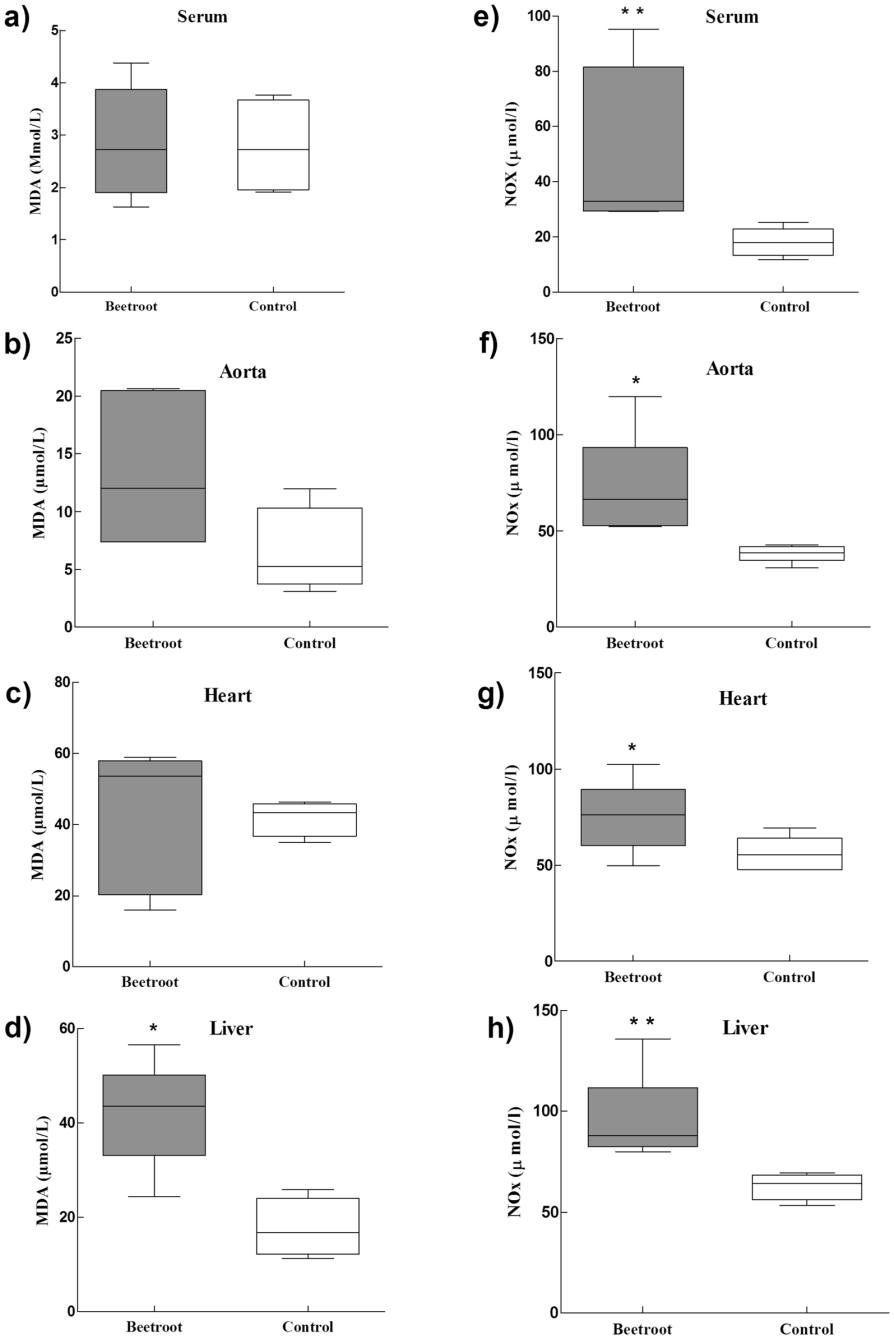
The effect of red beetroot juice on MDA (left side) and NOx (left side) in plasma, aorta, heart, and liver of studied groups. Red beetroot juice increased NOx content in plasma (**e**), aorta (**f**), heart (**g**) and liver (**h**) end of 6 weeks of study. There are 6 rats in each group. Statistical comparison between groups was made using a Mann–Whitney U test. MDA levels in plasma (**a**), aorta (**b**), heat (**c**), and liver (**d**) is illustrated at the end of the study. Red beetroot juice was fed for 6 weeks to 34 weeks old rats. There are 6 rats in each group. Statistical comparison between groups was made using a Mann–Whitney U test. Beetroot (red beetroot juice) vs. control (*P < 0.05, **P < 0.01)

The serum NOx level was dramatically increased in the RBJ group compared to the control group (p < 0.01, Fig. 3e). In addition, the NOx contents of aorta, heart and especially in the liver tissues of RBJ-treated animals were statistically higher than those in the control animals (p < 0.05, Fig. 3f–h).

Compared to the control group the serum, cardiac and aortic MDA levels did not change in the RBJ group (Fig. 3a–c). However, the liver lipid peroxidation level of the RBJ group was significantly higher than the control group (p < 0.01, Fig. 3d). However, to the best of our knowledge, its effects on pancreatic islets function have not yet been investigated. Hence, the present study was carried out to investigate the effect of red beetroot juice (RBJ) consumption on carbohydrate metabolism with a focus on islet insulin secretion and oxidative status in adult insulin-resistant rats (Additional file 1).

## Discussion

### RBJ reduced weight gain in adult rats

The mean daily intake of RBJ was about 60 mL (Table 1). Accordingly, the approximate NO_3_^–^ consumption was calculated nearly 100 mg/kg body weight. Our findings showed that the RBJ prevented weight gain in the treated group. It has been shown that inorganic NO_3_^–^ increases the number and activity of mitochondria, the consumption of oxygen in adipose tissue, and prevents weight gain in obesity-induced diets [19].

### RJB increased serum and tissue levels of NOx

The beetroot is rich in NO_3_^–^ sources [20, 21]. Previous studies have shown that even a single dose of beetroot juice increased serum NO_3_^–^ [22–24]. The present study also confirms the results of previous studies. Nitric oxide has two internal and external sources in the body. The intrinsic source is made of l-arginine amino acid produced under the influence of three isoforms of the NOS enzyme and its external source is derived from the consumption of nitrate-containing food. A recent finding suggests that dietary nitrate is metabolized to form NO and other bioactive nitrogen oxides [25]. It can be reduced to nitrite (NO_2_^−^) by NO_3_^–^ reductase activity of bacteria in the mouth. Then NO_2_^−^ in contact with gastric acid of stomach decompensates to NO by non-enzymatically fashion [11]. Therefore, after consumption of food containing NO_3_^–^, the level of NO metabolites increases. The possible beneficial effect of beetroot on cardiac performance, IR, vascular function, is related to the conversion of the NO_3_^–^ present in the RBJ to NO. The present study showed that RBJ consumption significantly elevated the liver, heart, and aorta NOx levels. Previous studies with different NO_3_^–^ contents of RBJ had been asserted NO_3_^–^ can stimulate the endogenous synthesis of NO in humans [26, 27] and rats [28]. Therefore, the NO_3_^–^ of RBJ in this study could stimulate NO formation in tissues and serum.

### RBJ did not affect normal blood pressure

RBJ consumption did not cause a significant change in blood pressure in rats. The result ties well with previous studies wherein the consumption of beetroot juice or its equivalent from NO_3_^−^ did not reduce blood pressure in healthy people or patients with hypertension [5, 22, 29, 30]. However, some other studies have shown that ingestion of beet juice contained 329–595 mg of NO_3_^−^ for 3–15 days reduced BP in healthy individuals [29, 31]. A systematic review of the effect of beetroot juice on BP has shown that the age of individuals affects the BP-lowering activity of beetroot. It has been shown that the antihypertensive effects of beetroot juice appear to be more pronounced in young people than in the elderly (over 65 years of age) [32]. This effect has been attributed to the reduced response of the vascular endothelium of the elderly to the NO_3_^−^ [33]. On the other hand, a systematic study and meta-analysis has shown that the RBJ has a dose and time-dependent antihypertensive effect (the lowest effect at a dose of 110–70 mL and the maximum effect at a dose of 500 mL daily) [34]. Otherwise based on this study, RBJ improved cell response to insulin. There is a traditional concept in which improvement of IR just leads to regulation of carbohydrates metabolism. However, insulin acts as pleiotropic hormone and exerts a multiple biological function, including wide range metabolic effect, ion and amino acid transport, NO synthesis, etc. In addition, insulin induces vasodilation through NO production [35]. In this study the RBJ consumption increased serum NOx as well as NOx content of aorta and heart. According to this view, RBJ can potentially reduce BP due to restoration of insulin delivery and preserving of NO availability.

### RBJ improved insulin sensitivity

The study results showed that RBJ improved insulin sensitivity in rats. Plasma insulin level decreased after glucose tolerance test, and the mean glucose level decreased by RBJ intake 2 h after the glucose injection.

It has been previously reported that 6 weeks beetroot juice ingestion decreased blood glucose levels in young healthy people [36]. Wootton-Beard et al., have reported that the use of beetroot juice prevented increased serum insulin levels following carbohydrate intake in healthy volunteers [37]. On the other hand, a clinical trial has shown that supplementation with either nitrate-rich beetroot juice (11.91 mM nitrate) or nitrate-depleted beetroot juice as placebo (0.01 mM nitrate) did not reduce plasma glucose in healthy adults (both young and old) [30]. In addition, Fuchs et al., reported that the use of a single dose (100 mL) of beetroot juice with 75 g of carbohydrates in insulin-resistant obese subjects with an average age of 61 years did not change the level of plasma glucose and insulin up to 3 h after use [38]. This inconsistence might be due to the type of administration (single vs. repeated). In agreement with many clinical studies, RBJ administration caused in improvement IR rats [36, 37, 39]. It has been previously demonstrated that NO_3_ improved glucose tolerance [40].

### RBJ decreased GSIS and diminished stimulatory effect NO on islet insulin release

To the best of our knowledge, there is no study investigated the effect of beetroot on islet insulin secretion. However, there is a study has indirectly investigated the effect beetroot consumption on β-cell insulin secretion. In the study, the healthy young and adolescent participants without overweight received 10% beetroot juice daily for 6 weeks, and then serum insulin and C-peptide were evaluated. Serum insulin and C-peptide had been significantly decreased [41]. In our study, also 5 weeks beetroot consumption decreased GSIS especially in supraphysiological glucose concentration. It is possibly attributed to the insulin-like potential of the phytochemical constituent of beetroot, mainly polyphenols of betanins, betanin and neobetanin [37]. A previous study has been proposed that RBJ through two probable mechanisms including suppression of glucose absorption and increasing insulin production/release can exerts its beneficial effects on glucose metabolism [37]. Our findings demonstrated that RBJ consumption could not stimulate insulin production/release in the pancreatic islets. This effects might be attributed to the beetroot ingredients such as NO_3_^– ^and NO_2_^– ^[20]. This study along with other study [28] showed that the NO increased insulin secretion as AG (NOS inhibitor) diminished islet insulin secretion.

### RBJ negatively influences liver function

RBJ consumption increased serum AST and liver MDA levels. These findings are contrary to some previous studies which have demonstrated hepatoprotective effects of beetroot [42, 43]. The high nitrate content of RBJ might be linked to liver damage. It has been previously reported that 60 days treatment with water containing 400 mg/L sodium nitrate significantly increased AST, ALT, and liver MDA in Sprague–Dawley albino rats [44]. Similarly, Anwar and Mohamed also reported that rats treated with water containing 500 mg/L for 28 or 42 days exhibited elevated levels of liver enzymes and kidney MDA level [45]. Considering the above mentioned evidence, it seems that the use of RBJ due to its high nitrate content may cause hepatic damage.

The present study has some limitations. One of the shortages of the present study is the lack of phytochemical analysis of RBJ. Also, in this study nitric oxide pathway were not completely assessed. Therefore, the assay of various enzymes like iNOS, eNOS and nNOS or their expression are recommended. The histopathological assay in different organs and assessment of some parameters related to ROS such as level of hydrogen peroxide and DCFDA assay were not conducted in the current study which should be considered in the future.

## Conclusions

RBJ consumption improves glucose metabolism in aged rats via increasing nitric oxide metabolites in an insulin-independent manner. However, future studies are needed to minimize the potential hepatic adverse consequences.

## Supplementary Information

**Additional file 1.** The effect of RBJ consumption for 4 weeks on hematologic and lipid profile was also investigated. The results confirmed that the RBJ didn’t show hematologic side effects also, it had no effect on lipid profile.

## Data Availability

The datasets are available from the corresponding author on formal and logic request.

## Abbreviations

RBJ: Red beetroot juice
IR: Insulin resistance
NO: Nitric oxide
BP: Blood pressure
IPGTT: Intraperitoneal glucose tolerance test
IP: Intraperitoneal
MDA: Malondialdehyde
HOMA-IR: Homeostasis model assessment of insulin resistance
GSIS: Glucose stimulated insulin secretion
HBSS: Hanks’ balanced salt solution
NOS: Nitric oxide synthase
AG: Aminoguanidine
TAG: Triacylglycerol
TC: Total cholesterol
LDL-C: Low-density lipoprotein
HDL-C: High-density lipoprotein
AST: Aspartate transaminase
ALT: Alanine transaminase
AUC: Area under the curve
FPG: Fasting plasma glucose
