# Supplementary material for: Effect of red beetroot juice on oxidative status and islet insulin release in adult male rats
Source: Diabetol Metab Syndr. 2022 Apr 23;14:58. doi: 10.1186/s13098-022-00830-z (PMC9034606; doi:10.1186/s13098-022-00830-z)
Supplement: Supplementary file 1 — Additional file 1. The effect of RBJ consumption for 4 weeks on hematologic and lipid profile was also investigated. The results confirmed that the RBJ didn’t show hematologic side effects also, it had no effect on lipid profile. [file 13098_2022_830_MOESM1_ESM.doc]

Additional file 1:

**Table S1.** Effect of RBJ on hematologic parameters and lipid profile

| **Variables** | **Groups** | |
| --- | --- | --- |
|  | **Control** | **Beetroot** |
| Cholesterol (mg/dl) | 68.50 ± 2.50 | 60.33 ± 3.04 |
| LDL-c (mg/dl) | 15.50 ± 2.50 | 15.00 ± 1.13 |
| HDL-c (mg/dl) | 35.00 ± 3.00 | 29.50 ± 2.20 |
| TAG (mg/dl) | 48.00 ± 14.00 | 39.50 ± 5.10 |
| WBC (*10^3^) | 5..00 ± 1.5 | 5.85 ± 0.38 |
| RBC (*10^6^) | 7.51 ± 0.025 | 7.03 ± 0.25 |
| PLT (*10^3^) | 823.0 ± 76.0 | 823.3 ± 83..41 |
| HCT (%) | 39.75 ± 0.05 | 42.43 ± 4.34 |
| Hgb (mg/dl) | 14.75 ± 0.05 | 13.73 ± 0.32 |

Statistical comparison between groups was made using a t- test, values are mean ± SEM, n= 6 each group. RBJ; red beetroot juice
